# Effects of sediment replenishment on riverbed environments and macroinvertebrate assemblages downstream of a dam

**DOI:** 10.1038/s41598-021-86278-z

**Published:** 2021-04-08

**Authors:** Izumi Katano, Junjiro N. Negishi, Tomoko Minagawa, Hideyuki Doi, Yôichi Kawaguchi, Yuichi Kayaba

**Affiliations:** 1 Aqua Restoration Research Center, Public Works Research Institute, Kawashima Kasada-machi, Kakamigahara, Gifu 501-6021 Japan; 2 Faculty, Division of Natural Science, Nara Women’s University, Kitauoya-higashimachi, Nara, 630-8506 Japan; 3 KYOUSEI Science Center, Nara Women’s University, Kitauoya-higashimachi, Nara, 630-8506 Japan; 4 Faculty of Environmental Earth Science, Hokkaido University, Sapporo, Hokkaido N10, W5060-0810 Japan; 5 Faculty of Advanced Science and Technology, Kumamoto University, 2-39-1 Kurokami, Chuo-ku, Kumamoto, Kumamoto 860-8555 Japan; 6 Graduate School of Simulation Studies, University of Hyogo, 7-1-28, Minatojima-minamimachi, Chuo-ku, Kobe, 650-0047 Japan; 7 Ecosystem Design Institute of Technology and Science, Tokushima University, 2-1 Minami-josanjima, Tokushima, 770-8506 Japan; 8 Water Environment Research Group, Public Works Research Institute, 1-6 Minami-hara, Tsukuba, 305-8516 Japan; 9 Departments of Architecture, design, civil engineering and industrial management engineering, Nagoya Institute of Technology, Gokiso-cho, Showa-ku, Nagoya Aichi, 466-8555 Japan

**Keywords:** Ecology, Freshwater ecology

## Abstract

Riverbeds downstream of dams are starved of sediment, impacting habitat structure and ecological function. Despite the implementation of sediment management techniques, there has been no evaluation of their conservational effectiveness; the impacts on high trophic level organisms remain unknown. This study examined the effects of sediment replenishment on riverbeds and macroinvertebrates in a dammed river before and after sediment replenishment. We evaluated the particle sizes of replenished sediments and the case material of a case-bearing caddisfly. We observed significant changes in macroinvertebrate assemblages before and after sediment replenishment, and between the upstream and tributary references and downstream of the dam. The percentages of Ephemeroptera, Plecoptera, and Trichoptera, and the number of inorganic case-bearing caddisflies downstream of the dam following sediment replenishment, were significantly higher than the upstream and tributary reference sites. The particle size of case materials used by case-bearing caddisfly corresponded to the size of the replenished sediment. Dissimilarity results after replenishment showed that assemblages downstream of the dam differed from upstream sites, although they were similar to the tributary sites. The dissimilarity between the tributary and downstream of the dam was the same as that between the upstream and tributary. Sediment replenishment was observed to reduce the harmful effects of the dam, and partly restore benefits such as increasing species diversity and altering community assemblages, similar to the effects of tributary inflows.

## Introduction

Sediment is an essential component of riverine ecosystems, and the balance of sediment transport and deposition significantly impacts aquatic biota^1^. However, water storage infrastructure, such as dams and reservoirs, interrupts the longitudinal continuity of sediment transport in river ecosystems through the construction of impoundments and subsequent flow regulation^2^. Small particle sediments such as sand and gravel, which accumulate in the upstream reaches of the dam and reservoir, decrease in the downstream reaches of the dam^3,4^. This reduction in small particle sediments in the riverbed alters downstream channel morphology^5^, detrimentally impacting aquatic habitat structures and ecological function downstream of the dam^6,7^. Such impacts include the loss of macroinvertebrate diversity^8,9^, the degradation of fish spawning beds^10^, excessive overgrowth of filamentous algae^11^, and a remarkably thick periphytic organic mat^12,13^. To address this downstream sediment deficit and mitigate morphological changes derived by sediment-starved waters, comprehensive sediment management strategies have been developed worldwide^14,15^.

Sediment replenishment (otherwise known as sediment augmentation) is a technique involving the artificial addition of bedload-sized sediment around the impoundment to the river channel downstream of the dam^16,17^. It is a technique that is applicable to dammed rivers worldwide due to its simplicity^18^. Sediment replenishment is an increasingly common practice in Japan^19,20^, as sediment production is high^21^, and sedimentation in impoundments is a serious problem for many small and intermediate-sized dams across Japan^22^. Sediment replenishment continues to be in the process of technical development, and there has been an accumulation of empirical data on its restorative efficacy for river morphology, such as its impact on channel incision^23^, and riverbed degradation^20,24,25^. However, there has been little evaluation of the ecological effectiveness of sediment replenishment on riverine ecosystems. Although there are a few reports stating that thick periphyton mats decrease following replenishment^26^, the efficiency of replenishment on high trophic level organisms, such as macroinvertebrates and fish, remains unknown.

A similar restoration technique, gravel augmentation (or gravel replenishment), has been widely used in regulated rivers in an attempt to construct riffles for salmon spawning in the United States (US)^5^. The gravel augmentation method involves relatively large riverbed materials, from gravel to boulder, and the materials are placed into a riverbed^2^. Its physical and ecological effects on the river ecosystem have been reported as an increase in the area of suitable riffles, mitigation of their physical conditions for salmon spawning^27^, and an increase in the biomass and diversity of fish^28,29^, and macroinvertebrates^30,31^. Macroinvertebrates in rivers play important roles in ecosystem functioning, for example, secondary production and the core of energy flow via the food web^1^. Staentzel et al.^31^ suggested the need for the assessment of post-restoration effects in macroinvertebrate structures for a better understanding of restoration consequences in regulated rivers. Although both techniques are premised on similar concepts, the effects of implementing these techniques on aquatic biota may be significantly different. The sediment size used differs significantly, and the effects of sediment load and material on organisms vary depending on the sediment size^32^. For example, net-spinning caddisfly *Hydropsyche* alters their behavior according to sediment particle size^33^. As it is impossible to apply the effects of gravel augmentation to sediment replenishment, it is important to investigate the effects of sediment replenishment on riverbed ecosystems, including the riverbed structure and benthic macroinvertebrate community. For case-bearing caddisfly, which utilizes fine sediments as their case materials^34,35^, the particle size structure of replenished sediments is likely to influence their use of case materials, and their fitness, abundance, and biomass under sediment replenishment.

The objective of this study was to examine the effects of sediment replenishment on the physical environment of the riverbed (i.e., water quality, drifted plankton, and benthic physical factors such as substrate coarseness, periphyton, particulate organic materials), and benthic macroinvertebrates (i.e., density, community composition, and diversity), downstream of a dam. These effects were examined along a dammed river before and after sediment replenishment. We hypothesized that downstream dam sites change before and after sediment replenishment; for example, the macroinvertebrate community would be restored by sediment supply via sediment replenishment, especially downstream of the dam, reducing fine sediment. If sediment replenishment has a sufficient effect on the benthos and the riverbed environment, there will be negligible differences between the reference and the downstream dam sites following replenishment. We also evaluated the particle size structure of replenished sediments and the case material utilized by the case-bearing caddisfly.

## Methods

### Study area

**Figure 1:**
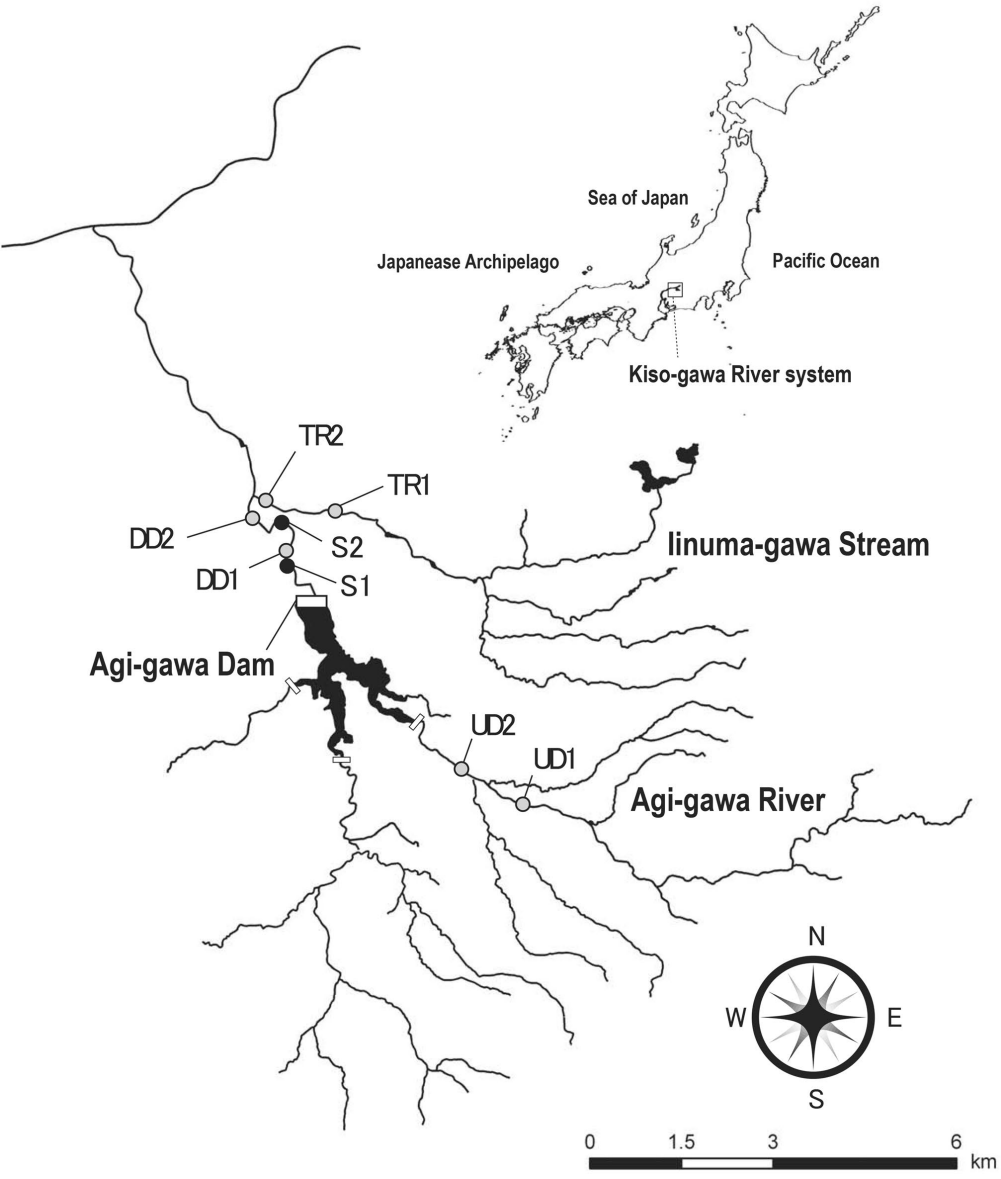
The study area shows six study reaches in three stream segments along the Agi-gawa River and Iinuma-gawa Stream, Gifu Prefecture, Japan. Gray circles denote reaches, which are numbered from upstream to downstream within each segment: UD1 and UD2 are upstream of the dam, DD1 and DD2 are downstream of the dam, whilst TR1 and TR2 are in the tributary. The two black circles denote the sediment replenished reaches (S1 and S2). The three small rectangles at the upstream ends of the impoundment are sub-dams, constructed to reduce the inputs of particulates to the impoundment. This map is based on the Digital Topographic Map 25,000 published by Geospatial Information Authority of Japan.

The study was conducted along the Agi-gawa River, a tributary of the Kiso-gawa River system in central Japan (35°23 42″–35°26 49″N, 137°25 12″–137°28 01″E; Fig. 1), with the Agi-gawa Dam (110 km from the river mouth, 418 m a.s.l.). The Agi-gawa River is a 3rd to 4th-order river with a naturally sand-rich bed derived from weathered granite that characterizes the local geology^36^. The Agi-gawa Dam (35°25 32″N, 137°25 55″E) had begun operations in 1990; it is a 102 m high rockfill dam with a catchment area of 82 km^2^, a storage capacity of 4.8 × 10^7^ m^3^, a mean depth of ~ 45 to 50 m at the dam site, and a hydraulic residence time of 71 days. Although three small sub-dams at the upstream end of the impoundment trap particulates, the sediment speed in the reservoir has been 1,000,000 m^3^ for 24 years. The dam serves multiple purposes, including flood control, industrial and urban water supply, and the maintenance of baseflow. Further information on the Agi-gawa Dam is available in Katano et al.^37^.

### Sediment replenishment and sampling sites

**Figure 2:**
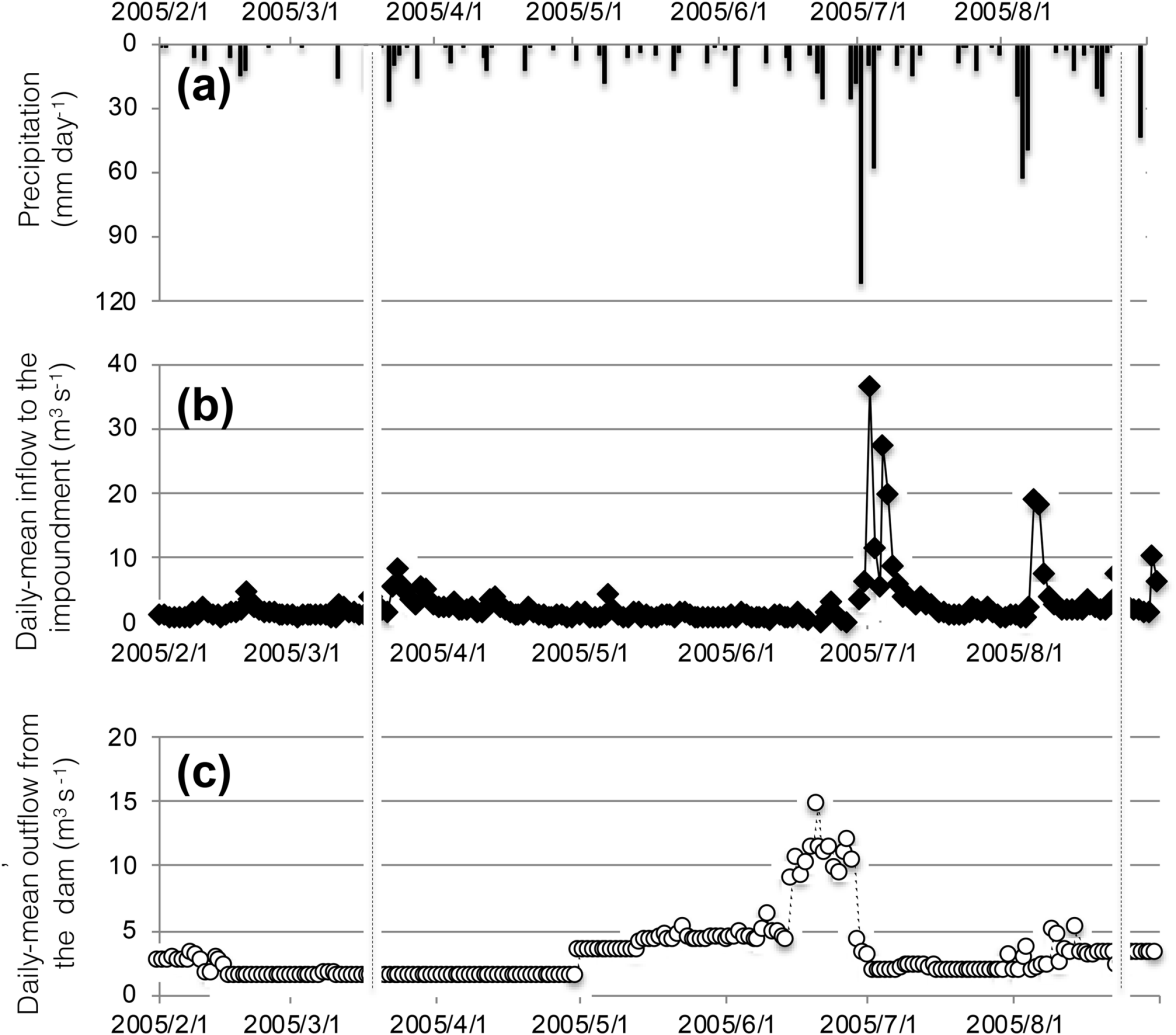
(**a**) Precipitation (mm·d) (**b**) mean inflow to the impoundment per day (m^3^·s^-1^); and (**c**) mean outflow from the Agi-gawa Dam per day (m^3^·s^-1^). The vertical broken line indicates the study period. Note that the *y*-axes for (**b**) and (**c**) have a logarithmic scale.

Sediment replenishment was undertaken 0.8 and 1.8 km downstream of the Agi-gawa Dam (S1 and S2, Fig. 1) on February 16 and 27, 2005. A total of 1,200 m^3^ of sediment (D50 ≈ 0.6 mm; mainly sand) was mined from the upstream sub-dams and transported to S1 and S2. We estimate that this constituted 0.086% of the annual sedimentation in the Agi-gawa Dam (e.g., in 2007, replenished sediment per year × 100/sedimentation in the reservoir). The sediment (800 and 400 m^3^) was replenished at high-flow banks in both sites. The replenished sediment was gradually washed during the high flows at the end of June (visual observation by dam administrators) (Fig. 2). We confirmed that this replenished sediment remained on both banks in March, and no sediments remained on both banks in early July.

Field sampling was conducted twice between March 15 and 18, 2005, prior to sediment flushing and between August 22 and 24, 2005, following sediment flushing [7 weeks after the end of the sediment drift out (Fig. 2)]. The later sampling date was scheduled to investigate the continuous effects (i.e., not immediate effects) of replenished sediment on the riverbed environment and macroinvertebrate assemblages before the replenished sediment had completely been transported further downstream from S1 and S2.

Three study segments (length: 1–2 km each) were selected: (1) upstream of the dam and impounded area (UD); (2) downstream of the dam (DD); and (3) in the tributary (TR). These sites were along a 6.0 km stretch of the Agi-gawa River and a 1.0 km stretch of the Iinuma-gawa Stream (catchment area = 24 km^2^); the latter is a tributary that flows into the Agi-gawa River 2.7 km, downstream of the dam (Fig. 1, Table 1). Each segment contained two study reaches (six reaches in total), and each study reach was 160 m long with several pool–riffle sequences; all reaches were > 300 m apart. DD1 and DD2 were located immediately downstream of the sediment-displaced banks (S1 and S2; 100 m upstream of DD1 and DD2, respectively). Measurements at the two reaches within the same segment were completed on the same day, and the reaches were surveyed in an upstream direction. The dominant land use along the study area was paddy fields, with sparse riparian forest.

**Table 1:** General characteristics of the three study segments and two seasons.

Variables	Seasons	Study segments and reaches					
		UD		DD		TR	
		1	2	1	2	1	2
Distance from the dam (km)		5.3	4.1	1.1	2.4	4.3 (1.6)	3.3 (0.6)
Elevation (m a.s.l.)		469	432	304	286	324	289
Catchment area (km^2^)		21.5	35.4	83.4	84.7	20.7	21.2
Slope (%)*		3.5	2.6	1.1	1.4	2.6	2.5
Discharge (m^3^ s^-1^)**	March	1.6 ± 0.3	1.4 ± 0.8	1.1 ± 0.4	1.5 ± 0.4	0.4 ± 0.1	0.5 ± 0.1
	August	2.2 ± 0.4	3.8 ± 0.8	3.6 ± 0.4	4.1 ± 0.4	0.7 ± 0.1	0.9 ± 0.2
Wetted width (m)**	March	10.3 ± 2.1	10.2 ± 1.8	8.4 ± 1.9	8.9 ± 1.6	4.5 ± 0.6	5.6 ± 1.2
	August	14.0 ± 0.8	12.5 ± 0.4	11.2 ± 1.0	10.9 ± 1.6	5.7 ± 1.0	6.4 ± 1.6

The distance of the tributary (TR) from the dam is the sum of distances from the dam to the tributary confluence, and from the confluence to the TR. Values in parentheses show the distance from the confluence. Data are shown as mean ± 1 SD (*n* = 6) where applicable.

*UD* upstream of dam, *DD* downstream of dam.

*Calculated from geographycal information system elevation data (50–3 resolutions; Geographical Survey Institute of Japan).

**Based on 6 transect lines across the channel at each study reach; flow discharge estimated from velocity at 60% of the depth and water depth at 5 equidistant points along each transect.

Although the most suitable reference site for DD is the DD prior to dam construction, we were unable to investigate the site prior to dam construction. Therefore, we treated the reference sites as sites that were less affected by the dam than DD on the present day. Katano et al.^37^ indicated that the difference between the TR and UD sites was smaller than that between DD and UD/TR sites in terms of biota and geology. However, UD was characterized by a wider channel and higher discharge than TR, due to differences in their catchment areas (Table 1). As we did not have a definitive reference, we treated both UD and TR as reference sites (see “Statistical analysis” section). Therefore, how DD in March and DD in August is different from UD and TR can be interpreted as the effect of sediment reduction.

### Physical environment and water quality

Six riffles were selected at each study reach, and a sampling location (50 × 50 cm quadrat) was established in the mid-channel area of each riffle. Prior to invertebrate sampling, physical environmental variables were measured.

Substrate coarseness was measured by gently floating a Plexiglas observation box (50 × 50 × 10 cm deep) divided into four grid squares (25 × 25 cm) on the surface water such that the grid had projected onto the streambed. The size of the substrate material was coded based on the intermediate-axis length: 1 = sand (particles < 2 mm), 2 = gravel (2–16 mm), 3 = pebbles (17–64 mm), 4 = cobbles (65–256 mm), and 5 = boulders (257–1024 mm). The percentage of each grid square covered by each coded category was measured, and substrate coarseness in the grid square was calculated as per Eq. (1):

1 $${\text{Substrate coarseness}} = \Sigma \left( {{\text{size category code }} \times \% \,{\text{covered by that category}}} \right)$$

The average substrate coarseness of the four grid squares was used to represent substrate coarseness at each sampling location. Water depth and current velocity at 60% of the depth were measured at the center and each corner of the quadrant with a ruler and an electromagnetic current meter (AEM-1D, Alec Electronics Co., Ltd., Kobe, Japan). The average values of the five velocities and depth measurements were used to represent the average velocity and depth at each sampling location.

Water quality [i.e., water temperature, electric conductivity, turbidity, and dissolved oxygen (DO)] was measured four times (every 6 h starting from 0600 h) near the upstream end of each reach to obtain diel changes. Water temperature and DO were measured with a thermometer and a DO meter (YSI-58; Yellow Springs Instruments, Yellow Springs, Ohio, USA), respectively, while conductivity and turbidity were measured with a water-quality probe (U-10; Horiba Ltd., Kyoto, Japan).

The bedload concentration was estimated by collecting and measuring transportable particles near the streambed at baseflow. Bedload sediment samples were collected using a handmade bedload trap (mouth opening = 20 × 30 cm, mesh size = 250 µm, catch bag length = 1 m), positioned at the upstream end of each study reach. The three traps were placed at equal intervals in a row perpendicular to the flow with the opening flush of the riverbed. The traps collected sediment for 1 h, whereby the current velocity at the center of the mouth opening was measured twice (at the beginning and end of the sampling) to estimate the water volume (m^3^) passing through the traps. Each bedload sample was later combusted in a muffle furnace (FO610; Yamato Scientific Co., Tokyo, Japan) at 550 °C for 4 h, and the inorganic fraction was determined using an electronic balance (AW220; Shimadzu Co., Kyoto, Japan). The bedload concentration (mg m^−3^) was obtained by dividing the mass of inorganic sediment by the water volume passing through the trap. A sample from reach DD1 in March was excluded due to contamination.

### Drifting plankton

Drifting plankton from reservoirs is characteristic of downstream areas of dams, which have a significant impact on benthic macroinvertebrates (e.g.^38^). Therefore, we also investigated the density of drifting plankton. Zooplankton were collected using two drift nets (mouth opening: 20 × 20 cm, mesh size: 250 µm, catch bag length: 0.8 m), anchored side-by-side perpendicular to flow and near bedload collection points. Samples were collected four times (every 6 h starting at 1800 h) over 20 min, and each sample was immediately preserved in 5% formalin. The water volume passing through the nets was calculated using the same method as that applied to the bedload traps. Zooplankton were identified to the lowest taxonomic level possible following Mizuno and Takahashi^39^ and Tanaka^40^, and counted under a dissection microscope (MZ12; Leica Microsystems GmbH., Wetzlar, Germany). The total drift density of zooplankton at each trap (number of individuals per m^3^) was determined by dividing the total number of zooplankton individuals by the water volume passing through the trap. Values from the two drift nets on each sampling occasion were averaged such that there were a total of four values for each reach.

For phytoplankton analysis, surface water was sampled in a polyethylene bottle (250 mL) four times (every 6 h starting at 1800 h), near each of the bedload collection points, and each sample was immediately preserved in 5% formalin. Well-mixed samples were placed in a counting chamber (Burker–Turk hemocytometer; ERMA, Tokyo, Japan). Phytoplankton were identified to the lowest taxonomic level possible using the taxonomic keys in Krammer and Lange-Bertalot^41^ and Hirose and Yamagashi^42^; they were counted under a light microscope (BX50; Olympus Co., Tokyo, Japan). This was used to determine the total drift density of phytoplankton (cells·mL^-1^).

### Benthic organic matter, macroinvertebrates, and periphyton

All bed materials were collected with a Surber sampler (frame size = 50 × 50 cm, mouth opening = 50 × 50 cm, mesh size = 250 µm, catch bag length = 1 m), to a depth of 20 cm at each sampling location. Immediately after collection, invertebrates and organic matter were brushed off substrates larger than pebbles (> 16 mm) and sieved through a 0.25 mm mesh sieve. Sieved samples and substrate material smaller than pebbles were mixed in a container and preserved in 5% formalin in the field.

The material in each container was later divided into two size fractions using 1-and 0.25 mm mesh sieves. To simplify the sorting process, all material retained in the 0.25 mm sieve was mixed and divided into 2^*n*^ subsamples (maximum *n* = 32) using a splitter (Idea Co., Tokyo, Japan), following the method described by Vinson and Hawkins^43^. All macroinvertebrates in subsamples in the 1 mm sieve were counted and identified to the lowest taxonomic level possible, usually to genus or species level using the taxonomic keys of Kawamura and Ueno^44^, Merritt and Cummins^34^, Kathman and Brinkhurst^45^, Kawai and Tanida^35^, and Torii^46^.

Macroinvertebrate taxa were also classified into five functional feeding groups (FFGs) according to Kawamura and Ueno^44^, Merritt and Cummins^34^, Kathman and Brinkhurst^45^, Kawai and Tanida (2005)^35^, and Torii^46^. FFGs were defined as collector-filterers, collector-gatherers, predators, scrapers, and shredders. If a species belonged to ≥ 2 FFGs, the number of individuals was apportioned across the FFGs. We also counted the number of burrowers (#burrowers), inorganic case-bearing caddisflies (#ICB), and net-spinners (#net spinners) of macroinvertebrate assemblages according Kawamura and Ueno^44^, Merritt and Cummins^34^, Kathman and Brinkhurst^45^, Kawai and Tanida^35^, and Torii^46^ (see Supplementary Table S1). This classification was carried out as such life-habit traits are important for surviving in a regulated river containing reduced quantities of sand and gravel on the riverbed^37^. The Chironomidae family was excluded in the life-habit analysis as they consist of various life forms. Once all invertebrates were removed, dry mass (mg m^−2^) and ash-free dry mass (AFDM, mg m^−2^) of benthic coarse particulate organic matter (BCPOM, > 1 mm), and benthic fine particulate organic matter (BFPOM, < 1 and > 0.25 mm) were obtained by drying in an oven at 60 °C for 1 day and combusting in a muffle furnace at 550 °C for 4 h. BCPOM and BFPOM were calculated based on the difference between the dry mass and the AFDM.

The total number of invertebrate individuals and the AFDM of BFPOM in each sample were estimated by multiplying by the corresponding 2^*n*^ value. The number of taxa and density of invertebrates in each sample were calculated as the sum of the values in both size fractions. Additionally, we determined Shannon's diversity index (*H*), Simpson's evenness index, and the percentage of Ephemeroptera, Plecoptera, and Trichoptera (%EPT)^47^. A sample from UD2 in March had been lost and therefore could not be included in the analyses.

Periphyton was sampled from cobbles adjacent to each sampling location. Periphyton was removed from a 5 × 5 cm area on the upper surface of each cobble with a toothbrush. Each sample was placed in a separate container with 250 mL of water. Within 24 h of sample collection, a subsample of the well-mixed content in each container was filtered using a glass-fiber filter (GF/C; Whatman Co., Maidstone, UK). Each filter was placed in a separate vial with 20 mL of 99.5% ethanol and stored in a dark refrigerator at 4 °C for 24 h. The extracted pigments were measured using a spectrophotometer (U-1800; Shimadzu Co., Kyoto, Japan), following the method of Lorenzen^48^.

### Analysis of case materials of an inorganic case-bearing caddisfly

We compared the particle size structure of replenished sediment, riverbed sediment, and case materials for case-bearing caddisfly. The replenished sediment was directly sampled in a 1 L polyethylene jar at the upstream replenished bank (S1) on March 16, 2005 (Fig. 1). Riverbed sediment was sampled at two stations; 100 m upstream of S1, and 100 m upstream of DD1 between August 22 and 24, 2005. At each station of the river, a metallic narrow cup (200 mL) with a lid was pushed into a vacancy between the cobbles, which had been randomly selected, and fine sediments (up to small gravel) in the vacancies were sampled by closing the lid underwater. Sampling was carried out three times (i.e., three different vacancies in the cobbles), and subsamples were pooled for measurement. The replenished and riverbed sediment was combusted at 550 °C for 2 h in a muffle furnace to remove organic contamination. Combusted samples were separated with eight sieves with a mesh size range of 0.075–9.5 mm (JIS A 1204). Each fraction was weighed, and the grain size accumulation curve of each type of sediment and its D_50_ were obtained.

In a macroinvertebrate sample at DD1 between August 22 and 24, 2005, ten individuals from two case-bearing caddisfly larvae, *Glossosoma* sp. and *Gumaga orientalis*, which were prevalent at DD1 during this period (see Results), were randomly selected from the formalin-fixed sample. The case was carefully removed from the larvae and combusted as described above for the replenished and riverbed sediment. The number of case material grains was measured using a dissection microscope.

### Statistical analyses

We described results based on two main assumptions: (1) the DD in March is the dam-affected reach (cf. unregulated reaches UD and TR), and (2) the changes in DD from March to August were mainly a result of sediment replenishment. In the statistical analyses, the *p* criterion (⍺) was set at 0.05.

To consider the effects of the segment, replicate reach, and season on variables, nested multivariate analysis of variance (MANOVA) was used to test whether any measured variables at the riffle scale differed between segments (UD, DD, and TR). Three segments and two replicate reaches were nested within each season (March and August) and segment (i.e., Season/Segment/Reach), whereby measurements within each reach were treated as subsamples. In the MANOVA, we also consider the interactions of the variances to interpret the interactions among the sampling segments and seasons to consider the independent effects on the factors.

To perform MANOVA, we assumed that temporal variability was greater than spatial variability within each reach for variables measured over 24 h (e.g., water quality), and the opposite would hold true for variables measured only once (e.g., macroinvertebrates). Therefore, subsamples within each reach were either spatially or temporally replicated, depending on the variable type. Temporal replicates (four samples collected every 6 h) were treated as a repeated factor (time factor). A nested MANOVA was used for variables quantified once at each location (e.g., macroinvertebrates), and nested repeated-measures MANOVA (rm-MANOVA) were used for variables quantified over a 24 h period at each reach (e.g., water quality). When a significant difference was detected by MANOVA with non-significant interactions, each variable was tested separately with a nested ANOVA for variable groups once at each location or the nested rm-ANOVA for repeated-measured variables, as appropriate for the particular variable. The risk of inflating Type 1 errors for the ANOVA was reduced using Bonferroni adjustments.

These MANOVA and ANOVA tests were conducted with R version 3.6.0^49^. The residuals of each variable in each MANOVA and ANOVA model were verified using the Shapiro–Wilk normality test prior to analyses, and normality was improved using arcsine(*x*) or log (*x* + 1) transformation when appropriate.

Tukey's multiple comparison test in a one-way ANOVA model (Season/Segment/Reach) was used for comparisons between segments. Any significant changes in values for variables from UD to DD were interpreted as the effects of the dam based on the assumption that conditions in UD and DD were similar prior to dam construction; this was because replenished sediment had not been supplied in March (see before). However, UD may be unsuitable as a reference site compared with TR as the former may be at least partly affected by the dam. This may particularly be the case for benthic invertebrates, such as the interruption of the upstream flight of adult females^50^. Therefore, UD and TR were treated as reference sites for reservoir and tributary effects, respectively. This was because both were unaffected by the dam, and sediment replenishment as tributaries may function as sites for resource recovery for the dam-affected mainstem of the river^37,51,52^, despite differing watershed areas. Therefore, the similarity of variables between the TR and UD sites was statistically confirmed such that they could be treated as reference sites. As such, the recovery from March to August could reliably demonstrate the effect of sediment replenishment. For example, although the value at DD differed from that at TR and/or UD in March, it was similar to that at UD and/or TR in August.

Multivariate analyses were conducted using the R "vegan" package version 2.5.6 to compare invertebrate assemblage structures between segments. Bray–Curtis coefficients based on species abundance were used to calculate a dissimilarity matrix, and dissimilarities between UD and DD, and between TR and DD in each season were tested using two-way ANOVA and Tukey post-hoc tests.

Macroinvertebrate assemblage organization in relation to environmental gradients was analyzed using redundancy analysis (RDA) with the "rda" function of "vegan" package. This was because the preliminary analysis using detrended correspondence analysis (DCA) showed that the gradient lengths of DCA were < 1.9. A gradient length of < 3 suggests that linear models, such as RDA, statistically outperform unimodal models, such as canonical correspondence analysis (CCA)^53^. Two matrices were included in the analyses: 1) density of each taxon × sampling site (response variable), and 2) environmental variables × sampling site (explanatory variables). Rare taxa (> 7 in the density rank in any of the samples in each season) were excluded from the RDA analysis. Environmental variables that were significantly different between seasons and/or segments by ANOVA were included in the RDA. In total, 71 samples with 17 taxa were used for the analyses. Permutation tests (1,000 iterations) were used to test whether eigenvalues from the RDA were significantly greater than those generated from a randomized matrix. The normality of the density of each taxon and the environmental variables were improved using arcsine(*x*) or log (*x* + 1) transformation.

### Ethics statement

Experiments in this study were conducted with approval from the Experimental Animal Ethics Committee, Nara Women’s University, Japan, carried out according to the Nara Women’s University Animal Experimentation Regulations, and the Act on Welfare and Management of Animals, Japan.

## Results

### Environmental variables

Across all study reaches, the discharge and wetted width in August were higher than those in March (Table 1). There were significant effects of season and segment observed for the environmental and biological variables measured multiple times, and the variables measured only once (MANOVA, *p* < 0.0001). The interaction effects between seasons and segments were detected for only two variables: zooplankton and phytoplankton (ANOVA, Table 2). However, the remaining nine and 13 variables differed significantly between seasons and segments, respectively.

**Table 2:** Mean (SD) values of environmental and macroinvertebrate variables at each study segment in each season, and the results of nested or nested repeated-measures analyses of variance (rm-ANOVA).

Variables	Seasons	Studey segments and reaches												ANOVA					
		UD				DD				TR				Season		Segment		Season[Segment]	
		Mean		SD	*n*	Mean		SD	*n*	Mean		SD	*n*	*F*	*P*	*F*	*P*	*F*	*P*
Water temperature (°C)	March	6.3	±	3.1	(8) b	7	±	1.4	(8) b	6.7	±	1.5	(8) b	815.2	**< 0.001**	2.0	0.590	0.2	3.273
	August	21.7	±	2	(8) a	23.6	±	0.7	(8) a	21.9	±	1.1	(8) a						
DO (mg L^-1^)	March	12.1	±	1.1	(8) a	12.2	±	0.2	(8) a	12.3	±	0.2	(8) a	844.7	**< 0.001**	0.2	3.437	0.1	3.671
	August	7.8	±	0.1	(8) b	7.6	±	0.2	(8) b	7.8	±	0.3	(8) b						
Electrical Conductivity (mS m^-1^)	March	3.1	±	0.4	(8) d	5.3	±	0.1	(8) ab	5.7	±	0.1	(8) a	131.5	**< 0.001**	548.0	**< 0.001**	3.4	0.175
	August	2	±	0.5	(8) e	5.4	±	0.3	(8) ab	4.2	±	0.2	(8) c						
Turbidity (NTU)	March	0.9	±	0.4	(8) b	0	±	0	(8) c	1	±	0	(8) b	40.3	**< 0.001**	17.3	**< 0.001**	0.4	3.205
	August	2.1	±	0.8	(8) a	1	±	0	(8) b	1.5	±	0.8	(8) b						
Depth (cm) *	March	26.8	±	7.8	(12) b	31.7	±	6.7	(12) ab	25.2	±	6.6	(12) b	7.8	0.168	11.5	**0.004**	6.6	0.077
	August	29.5	±	4.5	(12) b	36.8	±	14.5	(12) a	28.9	±	5.3	(12) b						
Velocity (cm s^-1^)	March	58.2	±	20.9	(12) a	37.5	±	17.1	(12) b	57.4	±	11.1	(12) ab	30.1	**< 0.001**	4.8	0.286	6.8	0.070
	August	78.5	±	22.6	(12) a	74	±	21.5	(12) a	64.8	±	21.5	(12) a						
Bedload flux (mg m^-1^ s^-1^)	March	10.2	±	10.6	(6) b	1.2	±	1.9	(5) b	3.1	±	2.6	(6) b	5.2	**0.03**	4.7	**0.019**	0.1	0.911
	August	274.6	±	285.5	(6) a	6.6	±	3.6	(6) b	20.8	±	14.5	(6) b						
**Drift**																			
Zooplankton (N m^-3^)	March	0.0	±	0.1	(8) a	145.2	±	197.3	(8) b	0.2	±	0.5	(8) a	5.1	0.24	14.6	**< 0.001**	11.3	**0.001**
	August	0.1	±	0.1	(8) a	37.6	±	46.0	(8) b	0.0	±	0.0	(8) a						
Phytoplankton (N. cells mL^-1^)	March	77.9	±	33.5	(8) a	420.6	±	244.5	(8) b	23.1	±	5.7	(8) a	13.5	**0.01**	74.6	**< 0.001**	8.5	**0.008**
	August	6.3	±	4.0	(8) a	1043.6	±	423.3	(8) b	58.6	±	59.1	(8) a						
**Substrate composition**																			
Sand (%)	March	7.2	±	4.6	(12) a	1.5	±	1.5	(12) c	6.5	±	4.3	(12) ab	1.5	4.109	12.2	**0.003**	0.4	11.751
	August	5.7	±	3.8	(12) ab	2.6	±	1	(12) bc	4.2	±	3.1	(12) abc						
Gravel (%)	March	20.7	±	10.9	(12) a	6	±	3.5	(12) c	17.4	±	6.3	(12) ab	2.8	1.852	25.3	**< 0.001**	1.5	4.154
	August	21.5	±	8.5	(12) a	9.9	±	4.7	(12) bc	21.8	±	7.1	(12) a						
Pebble (%)	March	25.7	±	7.8	(12) a	15.7	±	4.6	(12) c	25.7	±	8.3	(12) a	1.6	3.853	12.1	**0.003**	4.2	0.433
	August	19.6	±	8.8	(12) abc	16.8	±	7.2	(12) bc	23.6	±	7.5	(12) ab						
Cobble (%)	March	32.7	±	11.5	(12) cd	45.9	±	9.1	(12) ab	37.2	±	9.3	(12) bc	3.3	1.436	16.1	**< 0.001**	5.4	0.179
	August	37.2	±	11.1	(12) bc	48.6	±	11.3	(12) a	40.1	±	7.3	(12) abc						
Boulder (%)	March	13.6	±	15.4	(12) b	30.9	±	13.8	(12) a	13.1	±	13.4	(12) b	1.2	4.931	8.5	**0.023**	3.8	0.596
	August	16	±	15.8	(12) ab	22.1	±	15.1	(12) ab	10.3	±	12.2	(12) b						
Substrate coarseness	March	3.2	±	0.4	(12) b	4	±	0.3	(12) a	3.3	±	0.3	(12) b	0.4	9.862	26.8	**< 0.001**	1.3	5.335
	August	3.4	±	0.4	(12) b	3.8	±	0.2	(12) a	3.3	±	0.3	(12) b						
Chlorophyll *a* (mg m^-2^)	March	18.5	±	9.1	(12) a	87	±	87.7	(12) b	2.3	±	1.4	(12) a	9.5	0.081	11.1	**0.005**	3.8	0.613
	August	12.9	±	5.2	(12) a	13.1	±	8.8	(12) a	11	±	7.6	(12) a						
BCPOM (mg m^-2^)	March	9.3	±	5.1	(12) b	9.2	±	9.3	(12) b	17.2	±	13.8	(12) a	27.5	**< 0.001**	3.9	0.550	4.0	0.535
	August	5.3	±	2	(12) b	2.4	±	1.7	(12) b	4.7	±	1.7	(12) b						
BFPOM (mg m^-2^)	March	3.5	±	1.6	(12) ab	3.6	±	1.9	(12) ab	4.3	±	3.6	(12) ab	38.9	**< 0.001**	1.8	3.312	2.1	2.578
	August	2.4	±	1.4	(12) bc	0.3	±	0.2	(12) c	0.7	±	0.7	(12) c						

n = total number of subsamples in each segment. The effects that were significant with corrected α are denoted in bold font. Values of a variable labeled with the same letters are not significantly different (Tukey’s multiple comparison test under one-way ANOVA design).

*UD* upstream of dam and impoundment, *DD* downstream of dam, and *TR* in the tributary, *DO* dissolved O_2_, *BCPOM* benthic coarse particulate organic matter, *BFPOM* benthic fine POM.

In March, 13 environmental variables were significantly different in DD from UD and/or TR (Tukey’s post-hoc test, Table 2). In the 13 variables, nine variables in DD were significantly different for UD and TR. Turbidity, % sand, % gravel, and % pebble in DD were significantly lower than in UD and TR, while zooplankton, phytoplankton, % boulder, substrate coarseness, and chlorophyll *a* (periphyton) in DD were significantly higher than those in UD and TR. The remaining four variables in DD were significantly different from either UD or TR; electrical conductivity, velocity, and % cobble in DD were higher or lower than those in UD, while BCPOM in DD was lower than in TR. Electrical conductivity and BCPOM were significantly different between UD and TR, while there was no significant difference in velocity and % cobble between UD and TR.

In August, the 13 variables in DD had altered in four key aspects. First, % sand, % pebble, % boulder, and chlorophyll *a* in DD were not significantly different from UD and TR. Second, the velocity and BCPOM in DD had changed to not being significantly different from UD and TR. Third, turbidity in DD had changed to not being significantly different only with TR. In terms of conductivity and % cobble, which differed only with UD in March, they were now significantly different from UD and TR in August. The zooplankton, phytoplankton, % gravel, and coarseness in DD did not change statistically and still differed from UD and TR.

### Macroinvertebrate assemblage

**Figure 3:**
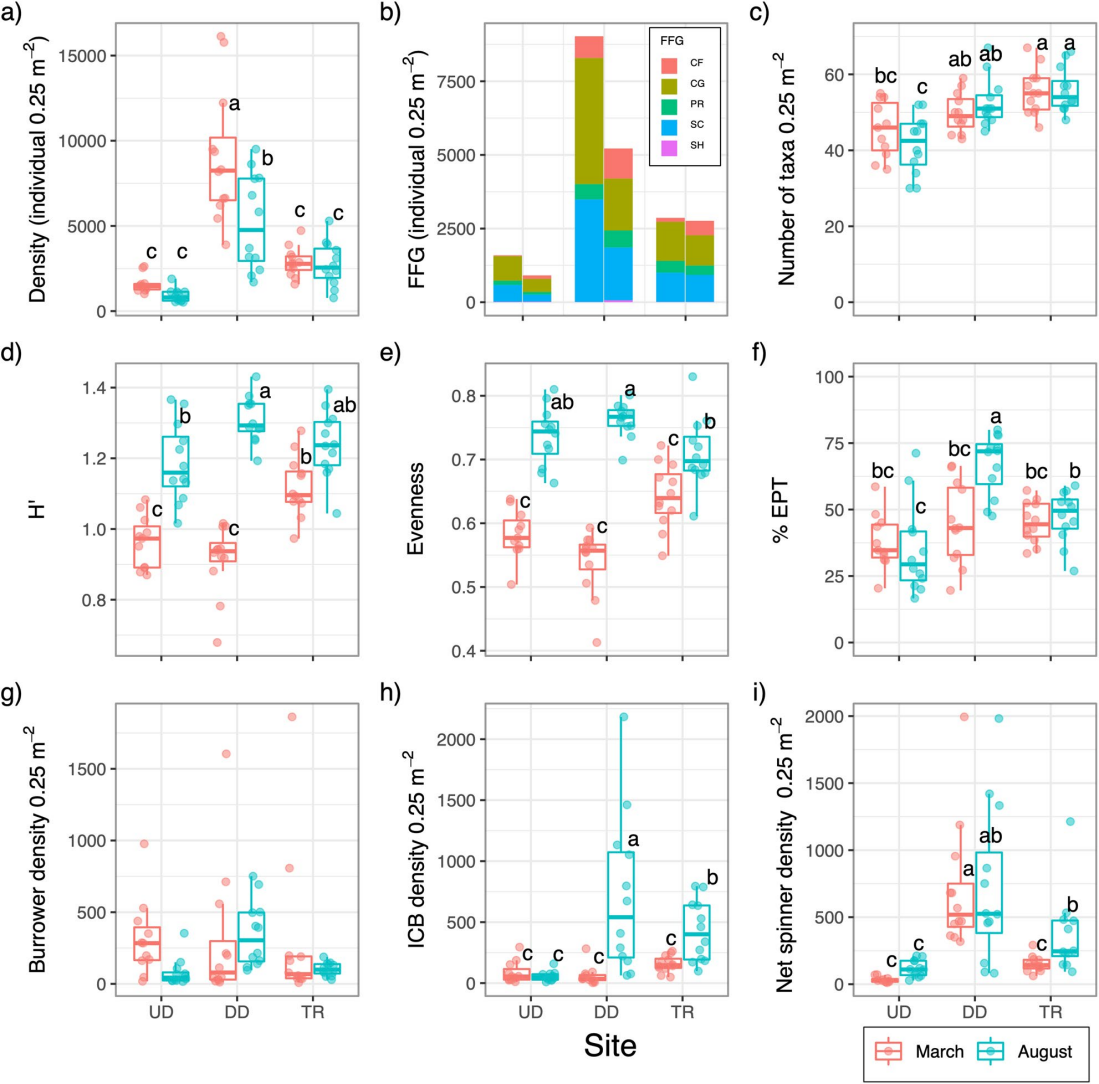
(**a**) Mean total density; (**b**) functional feeding group (FFG) density; (**c**) number of taxa; (**d**) Shannon diversity (*H*′); (**e**) Simpson’s evenness; (**f**) %EPT (Ephemeroptera, Plecoptera, Trichoptera); (**g**) density of burrowers; (**h**) density of inorganic case bearing caddisflies, and (**i**) net spinners in study segments and seasons. In the box plot, the bold line in the box indicates the median value and upper and lower limits of the box, and the whiskers indicate the first and third quartiles and ± 1.5 × interquartile range, respectively. The point-on-box plot indicates each data point. The same letters on the boxes were not significantly different between seasons and segments (one-way ANOVA and Tukey test, *p* < 0.05). *UD* upstream of dam, *DD* downstream of dam, and *TR* in the tributary.

A total of 266, 593 individuals from 220 macroinvertebrate taxa were collected (see the detail data in Supplementary Table S1). The mean density ranged from 521 to 16,126 individuals per 0.25 m^2^ (UD1 in August and DD1 in March, respectively; Fig. 3a). And the mean number of taxa of that ranged from 30 to 68 taxa (UD1 in August and TR2 in March, respectively; Fig. 3c). Among the FFGs, the abundance of the collector-filterer (CF) significantly increased in August and DD than in UD (Tukey test, *p* < 0.05, Fig. 3b). The interaction effects between seasons and segments were detected for *H’* and the number of inorganic case-bearing caddisflies (#ICB) (ANOVA, Table 3). There was no significant difference in the number of burrowers between seasons and segments.

**Table 3:** Summary of nested analysis of variance (ANOVA) results testing for effects of segment and season for variables related to macroinvertebrate assemblage structure.

Variables	ANOVA					
	Season		Segment		Season [Segment]	
	*F*	*P*	*F*	*P*	*F*	*P*
Taxonomic richness	0.1	13.914	20.5	**< 0.0001**	1.6	4.050
Density	14.5	**0.012**	68.5	**< 0.0001**	2.6	1.605
*H'*	152.7	**< 0.0001**	9.1	**0.016**	**8.8**	**0.019**
Evenness	185.6	**< 0.0001**	0.2	14.734	7.0	0.059
%EPT	8.1	0.143	23.5	**< 0.0001**	1.7	3.638
#Burrower *	2.2	2.704	1.0	7.007	1.2	5.738
#Inorganic case bearer	44.0	**< 0.0001**	18.1	**< 0.0001**	17.1	**< 0.0001**
#Net spinner	1.5	0.223	**22.3**	**< 0.0001**	0.57	0.566

Bold font denotes statistically significant effects after Bonferroni adjustments. *H'* = Shannon diversity; %EPT = the percentage of Ephemeroptera, Plecoptera, Trichoptera, FFG = functional feeding groups.

Tukey 3factors (per season).

*Not included chironomidae because genus of the family has various life forms.

In March, three macroinvertebrate variables significantly differed between DD, UD, and TR. First, density and #net spinners in DD were significantly higher than those in UD and TR (Tukey test, *p* < 0.05, Figs. 3a, i). Second, *H'* in DD was significantly lower than in TR (Fig. 3d), while *H*′ in TR was significantly higher than that in UD. There was no significant difference in the remaining five variables between UD, DD, and TR (Fig. 3c,e–h).

In August, three variables in DD were changed, as shown in Fig. 3. First, *H'*, which differed significantly between DD and TR in March, as no longer significantly different between these two sites and was higher in DD than UD (Fig. 3d). Second, density was still significantly different between the UD and TR (Fig. 3a). Lastly, the #net spinners did not statistically change with TR and were still significantly different between DD and UD (Fig. 3i). In addition, three variables in the other five variables also changed significantly. The EPT ratio and #ICB in DD changed to being significantly higher than UD and TR (Fig. 3f,h), and the number of taxa was higher in DD than UD (although not TR) (Fig. 3c). However, there was no significant change in evenness, which was still at the same levels in DD as those in UD (Fig. 3e).

**Figure 4:**
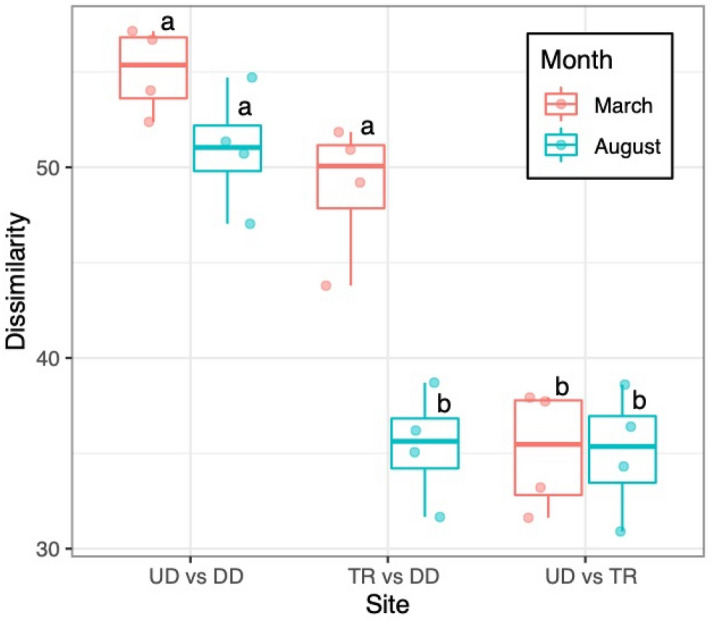
Bray–Curtis dissimilarity between segments in both seasons. The same letters on the boxes were not significantly different between seasons and segments (one-way ANOVA and Tukey test, *p* < 0.05). The bold line in the box indicates the median value and upper and lower limits of the box, and the whiskers indicate the first and third quartiles and ± 1.5 × interquartile range. The point-on-box plot indicates each data point. *UD* upstream of dam, *DD* downstream of dam, *TR* in the tributary.

Dissimilarities between the UD and DD, UD, and TR assemblages did not significantly change from March to August, while those between TR and DD changed significantly during that period (ANOVA, Segment *F* = 68.7, *p* < 0.0001, Season *F* = 21.9, *p* = 0.0002, the interaction *F* = 9.99, *p* = 0.0012, Tukey, *p* < 0.05, Fig. 4). Moreover, the dissimilarities between TR and DD in August did not significantly differ from that between UD and TR. The Chironomidae family (subfamilies Orthocladiinae or Chironominae) dominated most assemblages with the exception of DD in August, in which *Glossosoma* sp. dominated (Table 4). *Glossosoma* was a common taxon in all segments, particularly DD and TR in August (Table 4). An exception to this was DD in March, in which *Glossosoma* accounted for a small proportion of the assemblage (Supplementary Table S1). In this study, we identified the Chironomidae family as taxa, but further studies are needed to evaluate the Chironomidae genus or species for sediment supply.

**Table 4:** Density of numerically dominant taxa (top 5^th^ in rank) in the three study reaches at both seasons (individuals per 0.25 m^2^).

Reach	Rank	March					August				
		Taxa	Density	Cummulative % of total numbers of individuals	FFG	Life type	Taxa	Density	Cummulative % of total numbers of individuals	FFG	Life type
UD	1	Orthocladiinae gen. sp.	488.2 ± 117.4	30.8	CG, SC	AT, BR	Chironominae gen. sp.	144.7 ± 85.8	16.0	CG, CF	AT, BR*
	2	*Propappus volki*	306.4 ± 16.1	50.1	CG	BR	Orthocladiinae gen. sp.	138.7 ± 42.2	31.3	CG, SC	AT, BR*
	3	*Baetiella japonica*	230.3 ± 0.9	64.6	CG, SC	AT	*Propappus volki*	64.3 ± 54.2	38.4	CG	BR*
	4	Diamesinae gen. sp.	113.5 ± 21.0	71.8	CG, SC	BR	Naididae gen. sp.	62.6 ± 54.6	45.3	CG	BR**
	5	*Drunella sachariensis*	87.6 ± 0.0	77.3	PR	CR	*Antocha* spp.	51.4 ± 6.6	50.9	CG	AT
DD	1	Orthocladiinae gen. sp.	3489.6 ± 801.5	38.7	CG, SC	AT, BR*	*Glossosoma* sp.	623.2 ± 620.6	12.0	SC	CB
	2	*Uracanchella punctisetae*	1579.8 ± 33.2	56.2	CG, SC	CR	*Ephemerella* sp.	536.3 ± 425.7	22.2	CG, SC	CR
	3	*Baetiella japonica*	1047.3 ± 21.7	67.8	CG, SC	AT	Orthocladiinae gen. sp.	353.9 ± 269.6	29.0	CG, CF	AT, BR*
	4	Naididae gen. sp.	382.2 ± 1.0	72.0	CG	BR**	Chironominae gen. sp.	339.4 ± 57.3	35.5	CG, SC	AT, BR*
	5	*Hydropsyche orientalis*	254.8 ± 294.3	74.9	CF	NS	*Hydropsyche orientalis*	307.8 ± 231.2		CF	NS
TR	1	Orthocladiinae gen. sp.	776.3 ± 233.0	27.2	CG, SC	AT, BR*	Chironominae gen. sp.	574.4 ± 70.1	20.8	CG, CF	AT, BR*
	2	*Baetiella japonica*	306.9 ± 48.2	37.9	CG, SC	AT	*Glossosoma* sp.	387.6 ± 181.6	34.9	SC	CB
	3	Naididae gen. sp.	269.2 ± 278.0	47.3	CG	BR**	Orthocladiinae gen. sp.	231.9 ± 117.3	43.3	CG, SC	AT, BR*
	4	*Propappus volki*	230.2 ± 393.8	55.4	CG	BR*	Acarina fam. gen. sp.	187 ± 36.5	50.0	PR	CR
	5	*Drunella* spp.	190.4 ± 69.7	62.0	PR	CR	*Antocha* spp.	155.8 ± 72	55.7	CG	AT

Data are shown as mean ± 1SD (*n*).

*FFG* functional feeding groups. Life forms follow Kawai and Tanida (2005); *AT* attachers, BR* burrowers in deposit sediments, BR** burrowers in periphytic mat on substrata, *CR* Crawlers, *NT* net spinners, and *CB* case bearing caddisflies.

### Macroinvertebrate organization along environmental gradients

**Figure 5:**
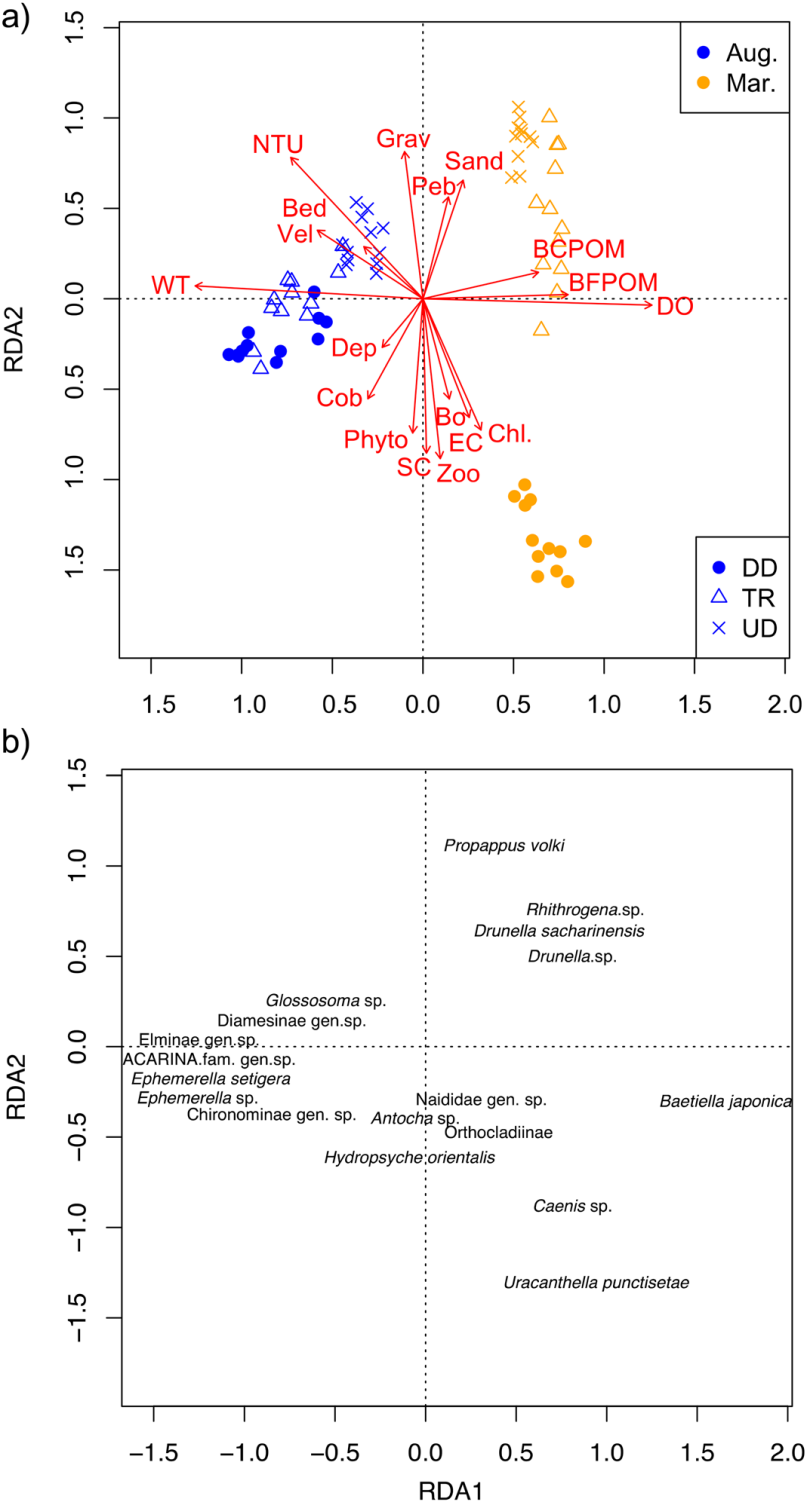
Biplot drawn from the redundancy analysis results showing the relationships between segment types, (**a**) environmental variables; and (**b**) dominant taxa. For the name of taxa, the position was jittered to avoid overplotting.

Three RDA ordination axes had eigenvalues of 0.365, 0.179, and 0.044 (Supplementary Table S2). The environmental variables significantly influenced macroinvertebrate assemblages (Permutation test; *p* = 0.001). Sampling locations within each season and each segment were clustered in the RDA plot, and the seasons and segments were clearly distinguished by the first two axes (Fig. 5a,b).

In terms of the sampling seasons, March and August could be clearly differentiated along axis 1. Axis 1 was positively correlated with DO, BFPOM, BCPOM, conductivity, % sand, % pebble, % boulder, chlorophyll *a,* and zooplankton, and negatively correlated with water temperature, turbidity, velocity, bedload, % cobble, depth, % gravel, substrate coarseness, and phytoplankton (Supplementary Table S2, Fig. 5). The former nine variables were relatively high in March, while the latter nine variables were relatively high in August (Fig. 5a, Table 2). Axis 2 was positively correlated with turbidity, % gravel, % sand, % pebble, velocity, bedload, BCPOM, water temperature, and BFPOM, and negatively correlated with zooplankton, substrate coarseness, phytoplankton, chlorophyll *a*, conductivity, % cobble, % boulder, depth, and dissolved oxygen (Fig. 5a, Supplementary Table S2). The former nine variables were relatively high in UD and TR, while most of the latter nine variables were low in UD and TR and high in DD (Fig. 5a, Table 2). There were clear differences between UD and DD along axis 2, representing the differences in terms of the dam effects manifesting in terms of riverbed materials and drift plankton (Fig. 5a). Unregulated TR reaches were also separated from DD along axis 2 in March, while the TR and DD reaches were plotted at the same locations in August (Fig. 5a). The RDA plots (Fig. 5a,b) and the dissimilarity results (Fig. 4) show that the structure of the macroinvertebrate assemblage differed between UD and DD in March; however, the DD assemblages changed to become similar to a TR assemblage in August, where this change is demonstrated by axis 2.

### Sediment compositions and case materials for case-bearing caddisfly

**Figure 6:**
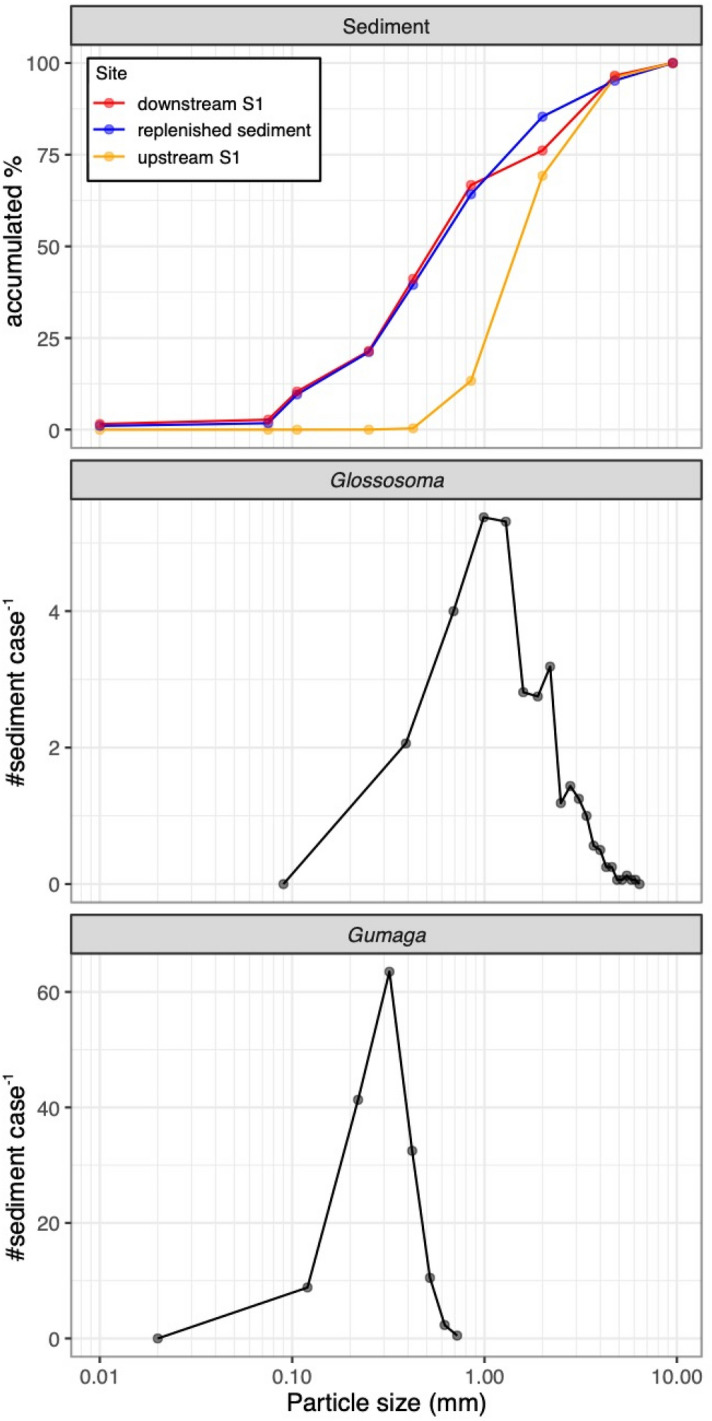
Frequency histograms of the mean sand grain distribution of the riverbed and replenished sediments, those in the cases of *Glossosoma* sp. and *Gumaga orientalis*. Note that the *x*-axes has a log_10_ scale.

Based on the grain-size accumulation curves, the replenished sediment mainly consisted of sand with a was 0.1–1.0 mm (D_50_ = 0.6 mm diameter), and the riverbed sediment composition was remarkably different from 100 m upstream and downstream of S1, the replenished station (Fig. 6a). The sediments upstream of S1 were lacking in fine sediment; for example, sediment < 0.46 mm in diameter (D_50_ = 1.6 mm). However, sediment downstream of the replenished station was similar to the replenished sediments, mainly consisting of sand (D_50_ = 0.6 mm).

The case materials of *Glossosoma* at DD in August consisted of 32.3 ± 7.67 grains (mean ± SD) that were between 0.13 and 5.79 mm in diameter (grain size = 1.62 ± 0.35 mm, Fig. 6b), and that of *G. orientalis* which consisted of 1411 ± 659 grains between 0.03 and 0.74 mm in diameter (0.27 ± 0.02 mm; Fig. 6c). In August, grain sizes used by case-bearing caddisflies were low in the riverbed sediment upstream of the replenished station, while they were abundant downstream (DD, Fig. 6a).

## Discussion

### Overview

We observed significant changes in riverine macroinvertebrate assemblages sampled at sites around the dam prior to and after sediment replenishment. While there was a partial difference in *H*′ in March, being significantly lower than TR, other variables such as species richness, evenness, %EPT, #burrowers, and #inorganic case-bearing caddisflies were not significantly different between UD, DD, and TR in March. This suggests that there were limited impacts of the dam observed in the relatively coarse-resolution community metrics for macroinvertebrates. Changes in macroinvertebrates (i.e., results in March) downstream of the dam were observed in terms of density and number of net spinners.

The effects of sediment replenishment and tributaries on macroinvertebrate assemblages, %EPT and #ICB in DD, were not significantly different between UD and DD in March, and were significantly higher than those of UD and TR in August. This is likely because case-bearing caddisfly *Glossosoma* was the most dominant. Dissimilarities in August showed that DD assemblages remained different from UD assemblages, although the dissimilarities between TR and DD significantly decreased to that between UD and TR, the latter two were unaffected by the dam. These results indicate that sediment replenishment is likely to somewhat reduce the negative effects of the dam (e.g., drastic increase in species density), while partly restoring the positive effects of the dam by increasing species diversity and altering the dominant species and assemblages.

### Effects of sediment replenishment on riverbed environments

Among environmental factors, % sand, % pebble, % boulder, and chlorophyll *a* tended to be sensitive to sediment replenishment in the dam-affected reach, as these variables in DD had changed significantly such that they were similar to UD and TR in August. However, % gravel and substrate coarseness in DD continued to differ from UD and TR in August; as such, both are likely to be factors resistant to sediment replenishment. Variables such as turbidity, electrical conductivity, velocity, % cobble and BCPOM, water temperature, DO, water depth, BFPOM, and bedload were those that relate to the natural regime of the effects of the dam, at least in this study.

The periphyton mat in DD was considerably different from that in the natural flow regime, which was under repeated transition by frequent disturbance. The periphyton mat in DD became thicker with high biomass as live algal cells such as hard filamentous green algae, such as *Cladophora* and *Spirogyra*, with low trophic values^11,54^ accumulate on dead algal cells^12,13^. These changes in the periphyton mat are likely to modify the food web of this river through grazer-periphyton interaction^55^. Indeed, there was a substantial decrease in the abundance of grazer Heptageniid mayfly^6^, grazer fish *Plecoglossus altivelis* decreased in density and in terms of their growth, downstream of the dam^56^. The detachment and transition of these periphyton mats is an objective of sediment replenishment^17^, via bedload sediment cleansing the periphyton mat^57,58^. This is because dam flushing in and of itself cannot detach thick periphyton mats and filamentous green algae^59,60^. The results from this study suggest that the cleansing effects of sediment replenishment on thick periphyton mats were effective for 2 months.

In addition to the cleansing effects of replenished sediment, the grazing effects by dominant grazing caddisfly *Glossosoma* are likely to have substantially contributed to a decrease in periphyton biomass. This is because *Glossosoma* larvae are powerful grazers on periphyton biomass and structure; the results showed that periphyton biomass remained at an extremely low level in instances where they have been dominant^38,61^. Although *Glossosoma* are widely distributed worldwide and in Japan^35^, to our knowledge *Glossosoma* and other ICB caddisfly have rarely been observed in the downstream reaches dams in Japan[e.g., 12,13,37]. Thus, sediment replenishment would be able to reconstruct biological interactions in the downstream ecosystem of the dam using these existing pathways.

### Effects on macroinvertebrate assemblages

The amount of bedload sediment in DD did not differ before and after sediment replenishment. The net-spinning caddisfly, *Hydropshyche*, recognized as the most intolerant taxa for bedload sediment^62^, was dominant in DD in both sampling seasons. Therefore, it is likely that the bedload sediment effect is negligible, at least under normal flows.

Sediment deposit was an important factor in determining macroinvertebrate density, richness, and distribution^63^, and had largely changed before and after the sediment replenishment. The macroinvertebrate density before sediment replenishment was highest in DD, in which the deposited sediment was low (1.5%); this is inconsistent with findings from previous studies^37,64^. With an increase in sediment deposits (from 1.5 to 2.6%) following sediment replenishment, the macroinvertebrate density decreased although it was still the highest. The decrease in macroinvertebrate density in DD is a pattern supported by Zweig and Rabení^65^, which is likely due to their habitat loss, as the deposit sediment filled vacancies that provide habitats and refugia for macroinvertebrates in coarse riverbed materials^66^. Gayraud and Philippe^67^ suggested that the density of many taxa, particularly with a body length of 5–10 mm, decreases due to habitat loss as a result of the filling of voids between coarse particles.

We demonstrated that *H*′, evenness, and %EPT increased with sand coverage, although these decreased in Zweig and Rabení^65^. Although these changes in this study were a result of the season, particularly for *H'* and evenness, they were at least partly caused by an increase in #ICB; this was lower in DD before the sediment replenishment. For FFGs, Rabení et al.^68^ found that scraper density was highest at approximately 0% sand coverage and gradually decreased with an increase in sand coverage (0–100%). Similarly, we found that with an increase in sediment coverage, scraper density decreased whilst #ICB, which consisted of some proportion of scrapers, increased in density as opposed to the unregulated reaches. As deposited sediment is commonly an abundant resource for macroinvertebrate taxa such as ICB^69^, marginally lower sand coverage is unlikely to impact ICB density. However, Statzner et al.^70^ revealed that sediment may be a limited resource during species life stages and riverbed areas, such as the pupal case materials of some caddisflies in high-velocity sites. Thus, the slight increase in deposited sediment in DD with extremely low sediment level (from 1.5 to 2.6% sand) is likely to meaningfully affect the occurrence of ICB, which was originally inhabited by abundant sand (UD: 7.2–5.7%, TR: 6.5–4.2%).

The ICB uses only suitable-sized particles for their case materials^71^, and they are sensitive to specific-sized particles. The particle size in the case materials of *Glossosoma* and *Gumaga*, following sediment replenishment, corresponded exactly to the size of the replenished sediment. The particle size for their case materials is likely to differ between taxa and life stages^71,72^. It is likely that there was an increase in the number of ICB taxa requiring particles that match in size with the replenished sediment.

Contrary to the ICB, burrowers do not have an apparent preference for particle size as they use the deposited sediment directly as their habitat^34^. In addition, burrowers included species using various depositional habitats (e.g., periphyton mat on stones), and it was difficult to separate taxa that inhabit the replenished sediment from other burrower taxa. Thus, burrowers are unlikely to respond to a slight increase in sediment deposits.

### Tributary inflow effects on macroinvertebrate assemblages and management implications

Tributary inflows drastically impact the downstream reaches of the dam, where previous studies have found improvements to the degraded environment and diversity of macroinvertebrates^37,64,73^. Katano et al.^37^ showed that the effects of tributary inflows on the DD environment were mainly caused by resupplying fine sediments; thus, sediment replenishment potentially has similar effects on macroinvertebrate assemblages following tributary inflows.

Tributary supplied sediment materials as well as organisms^52,74,75^, such as macroinvertebrates are likely to frequently drift from the tributary to the mainstem^37^, whilst adult females may fly upstream^76,77^. As species immigration may play an important role in tributary inflows, sediment replenishment in the river without tributary inflows may have less impact in terms of improvements to macroinvertebrate assemblages in DD than in rivers with tributary inflows. Further studies are needed to clarify these differences in the effectiveness of sediment replenishment.

In this study, we suggest that there are various effects of replenished sediment on environmental factors and macroinvertebrate assemblages. Our results show that a small sediment mass may improve riverbed environments and macroinvertebrate assemblages, at least in the short term. We suggest two implications for sediment replenishment. First, careful determination of the mass of the replenished sediment is required as this action may cause sedimentation when the sediment mass added is excessive. Sedimentation has been found to have a deleterious impact on macroinvertebrate assemblages^78–80^; for example, drifting macroinvertebrates at stations where boulders and cobbles dominated, increased when bedload sediment mass increased within a short time^81^.

Second, replenished sediment is likely to flush very quickly if the mass is too small. In this study, the total replenished sediment mass was 1200 m^3^, which represented 0.086% of the annual sedimentation in the Agi-gawa dam reservoir. This is considered a low level in terms of annual replenished sediment mass in Japan (100–25,300 m^3^^17^), and was less than the average annual ratio of sediment replenishment in Japan (0.1–12.9%^22^). These implications may be useful in terms of establishing a suitable sediment mass for sediment replenishment to avoid counterproductive impacts from sedimentation.

In this study, we demonstrate that sediment replenishment reduced the negative effects of the dam and partly restored the positive benefits by increasing species diversity and alterations to macroinvertebrate community assemblages, similar to the effects of tributary inflows. Based on the varying scales of sediment replenishment scales, such as the mass and substrate types, a suitable sediment replenishment procedure may be established to increase macroinvertebrate diversity and suitable community assemblages downstream.

## Supplementary Information

Supplementary Information 1.

Supplementary Information 2.
