# Supplementary material for: Effects of sediment replenishment on riverbed environments and macroinvertebrate assemblages downstream of a dam
Source: Sci Rep. 2021 Apr 8;11:7525. doi: 10.1038/s41598-021-86278-z (PMC8032774; doi:10.1038/s41598-021-86278-z)
Supplement: Supplementary file 1 — Supplementary Information 1. [file 41598_2021_86278_MOESM1_ESM.docx]

Supplementary Table S2. Summary statistics of the redundancy analysis (RDA) performed on macroinvertebrate assemblages and environmental variables in the 3 study segments during both seasons. Values for each environmental variable in the axis columns denote the correlation coefficients of each variable with the corresponding axis. DO = dissolved O_2_, BCPOM = benthic coarse POM, BFPOM = benthic fine POM.

|  | **Axis** |  |  |
| --- | --- | --- | --- |
|  | **RDA1** | **RDA2** | **RDA3** |
| Eigenvalue | 0.365 | 0.179 | 0.044 |
|  |  |  |  |
| Variables |  |  |  |
| Water temperature | -0.962 | 0.055 | -0.208 |
| Dissolved oxygen | 0.966 | -0.027 | 0.181 |
| Electrical conductivity | 0.196 | -0.502 | 0.789 |
| Turbidity | -0.557 | 0.599 | -0.326 |
| Depth | -0.171 | -0.205 | -0.229 |
| Velocity | -0.446 | 0.290 | -0.032 |
| Bedload flux | -0.249 | 0.221 | -0.718 |
| Zooplankton | 0.073 | -0.676 | -0.038 |
| Phytoplankton | -0.043 | -0.567 | 0.348 |
| % Sand | 0.170 | 0.502 | -0.048 |
| % Gravel | -0.077 | 0.623 | -0.218 |
| % Pebble | 0.107 | 0.431 | 0.221 |
| % Cobble | -0.232 | -0.422 | 0.198 |
| % Boulder | 0.111 | -0.423 | -0.113 |
| Substrate coarseness | 0.016 | -0.653 | 0.024 |
| Chl. a | 0.245 | -0.554 | -0.243 |
| BCPOM | 0.488 | 0.113 | 0.246 |
| BFPOM | 0.613 | 0.018 | -0.042 |
